# Phytochemical Profile and Pharmacological Activities of Water and Hydroethanolic Dry Extracts of *Calluna vulgaris* (L.) Hull. Herb

**DOI:** 10.3390/plants9060751

**Published:** 2020-06-15

**Authors:** Galyna Starchenko, Andriy Hrytsyk, Ain Raal, Oleh Koshovyi

**Affiliations:** 1 Pharmacy Department, Ivano-Frankivsk National Medical University, 76018 Ivano-Frankivsk, Ukraine; starchenkogalya@ukr.net (G.S.); grycyk@ukr.net (A.H.); 2 Institute of Pharmacy, Faculty of Medicine, University of Tartu, 50411 Tartu, Estonia; 3 Pharmacognosy Department, The National University of Pharmacy, 61002 Kharkiv, Ukraine; oleh.koshovyi@gmail.com

**Keywords:** *Calluna vulgaris* L., extract, phenolic substances, anti-inflammatory, neurotropic activity

## Abstract

According to the WHO data (2017), depression is the most predominant disease worldwide, with about 300 million people suffering from it, and bipolar disorder is the second most common. Therefore, it is relevant to research new medicines based on medicinal herbal raw materials with anti-anxiety and antidepressant effects. Common heather (*Calluna vulgaris* (L.) Hull.), a flowering plant of the Ericaceae family, is a promising species for research in this area. The aim of this study was to investigate the phytochemical profile and several biological activities of hydroethanolic dry extracts from the *C. vulgaris* herb. Nineteen phenolic substances were identified and quantified in the extracts by HPLC. The quantitative content of the basic groups of biologically active compounds was determined by spectrophotometry. Arbutin was dominant among the hydroquinone derivatives; chlorogenic acid among the hydroxycinnamic acids; rutin, hyperoside and quercetin-3-D-glucoside among the flavonoids; and (+)-gallocatechin and (-)-epigallocatechin among the tannin metabolites. The water and hydroethanolic extract were compared, the extract of *C. vulgaris* herb obtained with 70% hydroethanolic had the most pronounced anti-inflammatory, antimicrobial, anxiolytic, stress-protective, anti-anxiety and anti-depressant effects, and it is a promising substance for the development of new drugs or food supplements.

## 1. Introduction

Currently, the problem of anxiety is becoming more urgent due to changes in the social sphere, the intensity of lifestyles and other factors. According to the WHO data (2017), depression is the most prominent disease in the world, with about 300 million people suffering from it, and bipolar disorder is second most common. An estimated 6.3% of the Ukrainian population suffers from depression [1]. Special attention should be paid to the problems of a lack of an adequate choice of anxiety and panic disorders therapy, as well as a limited assortment of herbal medicines with anti-anxiety and antidepressant properties [2]. Therefore, it is relevant to research new medicines based on medicinal herbal raw materials with anti-anxiety and antidepressant effects.

Common heather (*Calluna vulgaris* (L.) Hull.), as a flowering plant of the Ericaceae family, is a promising species for research in this area. In particular, *C. vulgaris* is a pharmacopoeial plant in Germany and France [3], but not in the European Union or the Ukraine. Medicines based on biologically active substances (BAS) of the *C. vulgaris* herb or dry extracts of *C. vulgaris* (DECV) are not available in the Ukrainian pharmaceutical market [4].

In the Ukraine, this plant is widespread in Polissia, and to a lesser extent, in the forest zone of the Carpathians and Rostochchia-Opillia, but it is rarely found in the adjacent regions of the forest-steppe [5]. *C. vulgaris* grows on poor sandy acidic soils (pH = 3.5–6.7), which is due to the fact that it forms a symbiosis with underground fungi in the form of mycorrhiza, and thus transplanting an adult plant is impractical [6]. However, heather can grow in culture as an ornamental plant. There are more than 20 varieties of cultivated decorative heather [7,8].

The aim of this study was to investigate the phytochemical profile and some types of pharmacological activities of the DECV herb. The toxicity, anti-inflammatory, antimicrobial and neurotropic activity of the obtained dry extracts of *C. vulgaris* were studied for the first time. It has been shown that the most promising substance for creating new medicines from *C. vulgaris* is the extract obtained with a 70% ethanol solution.

## 2. Results and Discussion

### 2.1. Chemical Analysis

The extracts obtained are loose powders colored from light brown to dark brown, with a bitter taste and a specific odor. The yield of DECV from the plant material used was 10.42 ± 0.2% (extractant: purified water) and 16.79 ± 0.3% (extractant: 70% ethanol).

The hydroquinone glycosides, namely arbutin and methylarbutin, were identified and quantified (Table 1) by HPLC. The dominant substance in this group of BASs was arbutin, which is slightly more extractable by water than 70% ethanol (1.25% and 0.83%, respectively). Additionally, the total content of hydroquinone derivatives determined spectrophotometrically was higher in water DECV (10%, 51% and 7.86%, Table 2), which might be a promising substance for the development of diuretic and uroantiseptic drugs, especially arbutin and its metabolite hydroquinone [9,10]. Previously, arbutin was not detected in the plant material or a hydroethanolic (70% ethanol) extract of *C. vulgaris* by HPLC, surprisingly, even hydroquinone was not identified in *Arctostaphylos uva-ursi* [11].

In contrast, the content of hydroxycinnamic acids, flavonoids and tannins was higher in the hydroethanolic DECV. Chlorogenic acid was dominant among the hydroxycinnamic acids; rutin, hyperoside and quercetin-3-D-glucoside were dominant among the flavonoids; and (+)-gallocatechin and (-)-epigallocatechin were dominant among the tannin metabolites (Table 1). The content of flavonoids and polyphenols was twice as high in the hydroethanolic DECV (2.33% and 11.08%) than the water DECV (0.89% and 5.95%, respectively), so this substance may have pronounced anti-inflammatory and psychotropic activity. Additionally, Jurica et al. (2017) [9] showed that alcohol (methanol) was a better solvent for the extraction of phenolic compounds than water, and polyphenols are largely responsible for the antimicrobial activity of plant extracts.

In addition, it was previously shown that the basic BASs of *C. vulgaris* are phenols and their glycosides (hydroquinone, arbutin and methylarbutin), hydroxycinnamic acids, coumarins, flavonoids (kaempferol, quercetin, astragalin, hyperoside, herbacetin, luteolin and isoquercetin), tannins and their metabolites, and terpenes (α-pinene and β-pinene) [12,13,14,15]. It is a valuable source of BAS that has anti-inflammatory, antibacterial, sedative and anti-anxiety activities [16,17,18]. This makes it a promising plant for the development of new drugs or at least food supplements. From the other side, these compounds are rather common in many plant species but their qualitative and quantitative content in *C. vulgaris* is unique.

Spectrophotometric methods of analysis are convenient and appropriate for the development of methods for standardization of DECV as they are accessible, easy to perform and do not require high-value devices and consumables. The results of spectrophotometric analysis showed the same tendency as the HPLC method regarding the content of the basic groups of BASs in the DECV and will be used in the development of regulatory documentation.

### 2.2. The Pharmacological Activity of the DECV

#### 2.2.1. Determination of Acute Toxicity

The results of the study of acute toxicity of the water and hydroethanolic DECV showed that oral administration of the extracts at a dose of 6000 mg/kg did not cause death; the overall condition of the animals of the experimental groups was satisfactory; no visual changes compared with the control group were observed; all of the rats had a normal appetite; the color of the skin and mucous membranes was within normal limits, and their fur was smooth and shiny; body temperature and tissue turgor were unchanged; respiratory distress and seizures were not detected; but, a decrease in protective reflexes and fearfulness of the animals was observed. There was no change in the color of the urine and feces; the frequency of bowel movements also did not change. There was no change in the above characteristics both during the first day and throughout the observation period. The results of the toxicological studies indicate that the DECV are practically non-toxic according to the value of LD_50_ because their lethal dose could not be determined by intragastric administration. Thus, all test substances can be classified as the V toxicity class. To the best of the authors’ knowledge, data on the acute toxicity of *C. vulgaris* have not been published previously.

#### 2.2.2. Determination of Anti-Inflammatory Activity

**Figure 1:**
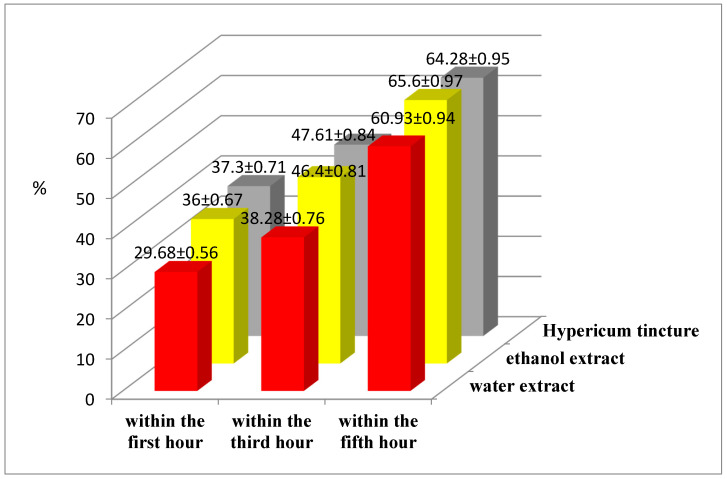
Anti-exudative activity of *Calluna vulgaris* herb extracts.

The results of the studies (Figure 1) indicate that the DECV showed anti-inflammatory activity. Compared to the control, an anti-exudative effect of DECV was observed within the first hour after the start of treatment and reached its maximum values at the third and fifth hour, depending on the solvent of the extract. The hydroethanolic DECV showed the highest anti-inflammatory activity among all tested extracts.

During the first hour of the experiment, the most pronounced anti-inflammatory activity was shown by the reference drug (Hypericum tincture), which reduced the oedema by 37.30% compared to the control. However, at the third and fifth hour of the experiment, the anti-exudative activity of the reference drug decreased slightly compared to the hydroethanolic DECV. In the group of animals treated with this extract, the volume of the rat paw decreased compared to the control group by 36.00% within the first hour of the experiment, by 46.4% at 3 h and by 65.6% at 5 h after the phlogogenic agent injection. 

After the experiment, all animals had peripheral blood taken from their tail vein and the content of hemoglobin and the number of red blood cells and leukocytes were determined (Table 3). It is a well-known fact that one of the indicators of an inflammatory process is leukocytosis. According to the data given in Table 3, the number of leukocytes in the control group of animals increased by 1.47 times. The use of all investigated substances in various ways contributed to the reliable normalization of the number of leukocytes, and at the end of the experiment, the indicators in the peripheral blood of the tested animals were brought to the level of the untreated controls. 

Thus, the results of the study indicate that both DECVs have anti-inflammatory activity. The hydroethanolic DECV had the most pronounced anti-exudative activity; it reduced the inflammatory reaction by 65.60% and had approximately the same effect as the reference drug (*Hypericum tincture*). This is consistent with Villanueva-Bermejo et al. (2019) [19], who showed a carbon dioxide extract using ethanol as a co-solvent showed a certain anti-inflammatory effect.

#### 2.2.3. Determination of Antimicrobial Activity

As a result of the conducted studies, it was established that the DECV inhibited the growth of microorganisms and had a bacteriostatic effect against *Pseudomonas aeruginosa* (the zone of inhibition of microorganism was 8–9 mm), *Escherichia coli* (8–10 mm), and *Proteus vulgaris* (6–7 mm), and partially against *Staphylococcus epidermidis* (0 mm for water and 7 mm for hydroethanolic DECV). The hydroethanolic DECV had the most pronounced antimicrobial activity against all tested microorganisms and had approximately the same effect as the reference substances ampicillin (0–14 mm) and oleandomycin (0–5 mm), if we exclude their activity against *S. aureus* and *S. epidermidis.*

Kakouri et al. (2017) [20] reported that hydroethanolic extracts rich in flavonoids and other phenolic compounds did not showed remarkable activity against the microorganisms mentioned above. On the other hand, the hydroquinone glycoside arbutin displayed relatively strong antibacterial activity against *S. aureus* but was absent in both DECVs containing arbutin [10]. According to Jurica et al. (2017) [9], the antimicrobial activity of leaf extracts of *Arbutus unedo*, other than arbutin itself, could be related to the presence of phenolic acids such as cinnamic, ferulic, and caffeic, but their content in both DECVs studied by us is not very high (Table 1). Arbutin also showed antibacterial activity against *P. aeruginosa* and *E. coli* (MIC > 25.6 mg/mL) in other study [9].

#### 2.2.4. Determination of Neurotropic Activity

**Figure 2:**
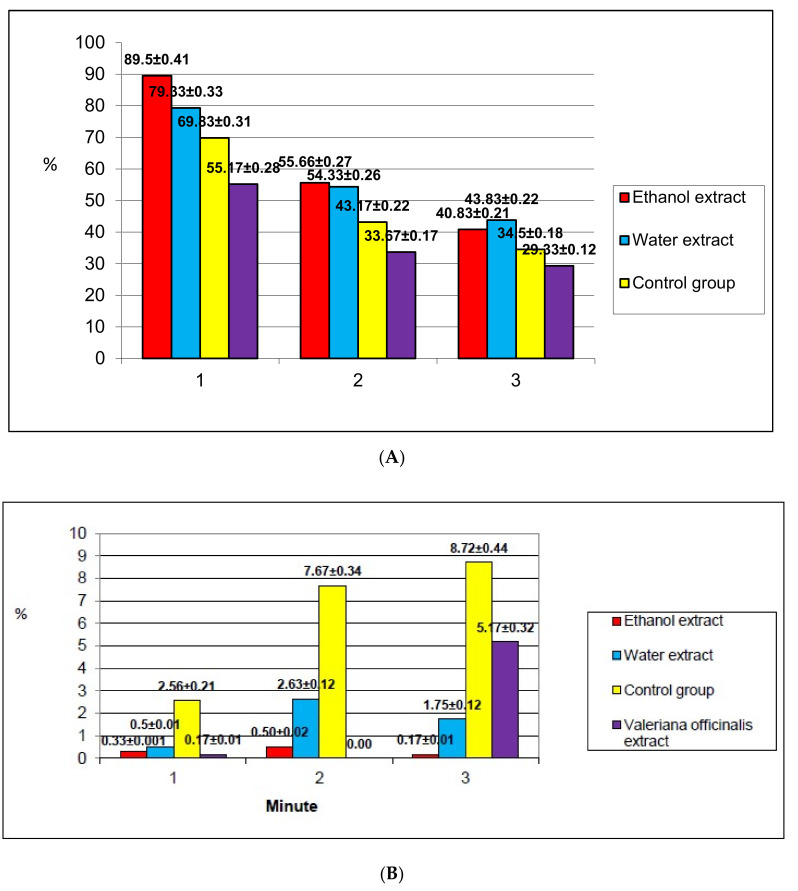
Comparative characteristics of the effect of investigated extracts on mice in the “Open Field” test. (**A**) Effect on the orienting-research behavior of mice. (**B**) Effect on the level of anxiety of mice.

The results of the study of the behavioral responses of mice in the “Open Field” test indicated a significant effect of DECV on the motor activity of animals (Figure 2). The novelty stress showed the greatest impact on the orienting-research behavior of mice in the “Open Field” arena within the first minute of the experiment. *C. vulgaris* extracts activated the locomotor behavior of mice due to their increased psycho-emotional excitation. The total motor activity increased mainly due to the large number of horizontal movements. Grooming increased significantly compared to the control in the group of mice treated with hydroethanolic DECV. Some authors have described grooming as an “indicator of comfort” for animals [21,22]. The latent period of exit from the center of the arena was not significantly different for all studied groups. The fading indicator in the “Open Field” test was of great interest. The hydroethanolic DECV did not cause fading of the animals at all, and the water DECV had approximately the same effect as the *Valeriana officinalis* extract.

Thus, both the water and hydroethanolic DECV activated the exploratory behavior of mice under the conditions of novelty stress without inhibition of their orienting-research activity. *Valeriana officinalis* extract reduced vertical motor activity and grooming, which means inhibition of orienting-research behavior.

#### 2.2.5. Determination of Anti-Anxiety and Antidepressant Activities

The “Elevated Plus Maze” test. The investigated DECVs influenced the level of anxiety, fear and perception caused by the novelty of its environment, its reaction to the height and to the illuminated open space (Table 4). In the analysis of the results, special attention was paid to the group of animals that were administrated hydroethanolic DECV. The total number of appearances of animals of this group in the dark chamber was 1.30 times higher compared to the control. The total time spent in the dark branches of the maze was determined in seconds. The mean value for animals treated with hydroethanolic DECV was the lowest (103.29 s) among all tested substances. The total number of appearances in the central location increased by 1.25 times compared to the control. The total number of appearances of animals in the illuminated branches of the maze increased by 1.33 times compared to the control.

The total time of staying in the illuminated branches was determined in seconds and was the highest for the animals of this group (138.43 s). The total time of staying in the central location was calculated in seconds and was the highest for the animals that were administered extract 2 (56.86 s). The total number of cases when the animals looked down from the maze was calculated to evaluate the risk of the animals. This indicator was the highest in this group of animals (19.86). 

Thus, the administration of the hydroethanolic DECV activated the orienting-research behavior of the animals and showed an anti-anxiety effect, which was determined by the large number of movements of animals in the illuminated branches of the maze and the number of cases of looking down. Animals in this group preferred to stay in the illuminated branches and in the center of the maze, which could be interpreted as a stress resistance. The large number of cases of looking down meant that the animals were not afraid to explore all ways of leaving the maze. The large number of appearances in the dark branches and the shortest time of staying there may indicate the high motor activity of the animals and favoring the illuminated branches of the maze. Considerable attention should be drawn to the number of appearances of the animals in the illuminated branches. This indicator was the highest during all 5 min of the experiment in the group of animals that were administrated hydroethanolic DECV.

The “Porsolt Forced Swim Test”. The antidepressant effect of the investigated DECV was evaluated by their ability to reduce the time of immobility and time of passive swimming of animals, and to increase the latent period of the first “fading” and the time of active swimming. In the conditions of the “Porsolt Forced Swim Test”, the administration of the tested extracts showed an antidepressant effect on the tested animals (Table 5). Thus, on the first day of the experiment, the latent period of the first “fading” of the animals treated with extract 2 was 1.18 times shorter compared to the control. The latent period of the first “fading” was extended on the third day of the experiment and lasted 1.10 times longer compared to the control. On the fifth day of the experiment, the latent period of the first “fading” of animals was reduced and lasted 1.22 times less compared to the control. The total time of active swimming of the animals of this group on the first and third days of the experiment was the longest (190.29 s and 99.57 s). On the fifth day of the experiment, the total active swimming time was 1.79 times shorter compared to the control. On the third day of the experiment, the time of total active swimming of animals treated with extract 1 was 1.17 times longer compared to the control.

Therefore, the hydroethanolic DECV exhibited antidepressant activity on the first and third days of the experiment, as evidenced by the longest period of active swimming and the shortest time of immobility of the animals. The time of passive swimming of the animals that were administered hydroethanolic DECV was the shortest on the first day of the experiment.

The “Cube” test. The anxiolytic effect of the investigated extracts was evaluated by their ability to increase the number of approaches of animals to the cube and the total time of the cube examination, as well as to reduce the latent period of the first approach of the animal to the cube. In the conditions of the “Cube” test, the administration of both DECVs to the animals affected the level of their anxiety and fear of a new object in their home cage (Table 6). The latent period of the first approach to the cube was the shortest in the group of animals receiving extract 2 (0.46 s). In this group of animals, both the number of approaches to the cube (5.00) and the total time of the cube examination (14.57 s) were the highest. 

It is interesting to mention that *C. vulgaris* is known in Estonian ethnomedicine for its sedative and anti-diabetes activities, but it does not have the anti-cancer activity found in the other 44 species [23,24,25]. *Hypericum perforatum, Leonurus cardiaca* and *Piper methysticum* are well known natural antidepressants and/or anxiolytics, and also the species *Epimedium* L. genus (Berberidaceae) belonging to the Chinese pharmacopoeia possesses antidepressant activity [26,27,28]. Their BASs are totally different, and also different than the *Valeriana* genus. Dhiman et al. (2020) [29] found that *Valeriana jatamansi* possesses sedative and antidepressant properties and is an alternative choice among existing drugs like benzodiazepines to treat insomnia. 

Additionally, *C. vulgaris* seems to be promising species to treat these health problems. The water and hydroethanolic DECVs increased the number of approaches of the animals to the cube, the total time of the cube examination, and reduced the latent period of the first approach to the cube, which indicates the activation of orienting-research behavior of animals and the presence of an anti-anxiety effect.

#### 2.2.6. Discussion of the Results

Considering the chemical composition of the obtained extracts, we can conclude that the content of hydrocinnamic acids and flavonoids in the hydroethanolic DECV are, respectively, in 1.34 and 2.61 times more than the aqueous one, and the sum of phenolic compounds, in general, is 1.87 times more. The extracts contain a high content of flavonoids—rutin, hyperoside and quercetin-3-D-glucoside—which also prevail in motherwort grass *Leonurus cardiaca* and preparations based on it [26,30,31], which extent determines the effect of extracts on the nervous system. There are more hydroquinone (1.33 times) derivatives in the aqueous extract which determines the activity of the extracts to different groups of microorganisms [8,10,11]. Phenolic compounds have anti-inflammatory activity [19,32,33], and as there are more of them in the hydroethanolic DECV, it is more active. In general, a greater amount of phenolic compounds in the hydroethanolic extract and it determines its advantages. The experiments showed that the hydroethanolic DECV possess anti-inflammatory, antimicrobial, anxiolytic, stress-protective, anti-anxiety and anti-depressant effects due the optimal concentration of ethanol (70%) used by us.

## 3. Materials and Methods 

### 3.1. Plant Material 

The aerial parts of *C. vulgaris* for the production of DECV were harvested in 2018 on the outskirts of the village Rozsil’na, Bohorodchany district, Ivano-Frankivsk region (geographical coordinates: 48.77861 N, 24.37901 E) in the flowering phase of *C. vulgaris.* The herbs were identified and harvested under the guidance of Professor Hrytsyk A. R. from the Ivano-Frankivsk National Medical University (IFNMU) according to the botanical catalogue [34]. The raw plant material was dried for 10 days at room temperature in a ventilated room. The voucher specimens are stored at the Department of Pharmacy, Ivano-Frankivsk National Medical University, Ivano-Frankivsk, Ukraine (No 34-51).

### 3.2. Preparation of Extracts

The plant material was ground to a particle size of 0.25–2.5 mm and loss during drying was tested [31,32]. To obtain the aqueous extract from the herb of *C. vulgaris*, 500 g of ground plant material was extracted with 5000 mL of purified water by the method of bismaceration at a temperature of 80–90 °C for 30 min. The extract was filtered, and the residue of the raw material was extracted under similar conditions twice more. The extracts were combined and filtered (Water DECV).

To obtain the aqueous-alcoholic extract from the herb of *C. vulgaris*, 500 g of ground plant material was extracted with 5000 mL of 70% hydroethanolic by the method of bismaceration, under similar conditions as for the aqueous extract. The hydroethanolic was distilled off under vacuum in a rotary evaporator. After the hydroethanolic distillation, the total volume of the extract was brought to the original volume with water (hydroethanolic DECV).

The extracts were frozen in alcohol baths at the temperature of alcohol not higher than –40 °C for 30 min. After that the vials with frozen extracts were placed into a freezer maintained at a temperature not higher than −30 °C, and stored before loading into the sublimation dryer for 12 h. Drying of the extracts was carried out in a sublimation dryer of KS-30 type. In the initial drying period, the pressure in the sublimator was reduced from 1 × 10^−1^ to 1 × 10^−5^ mm Hg and the temperature of the frozen extracts from −35 to −50 °C. After 2–2.5 h, the heating was turned on and after 12–16 h, a constant temperature increase was conducted. The temperature of the product in the final drying period was not higher than +40 °C. The total duration of drying was 28–32 h.

Standardization of the DECV was performed according to the requirements of the SPhU [35], namely description, identification, residual amounts of organic solvents (using gas chromatography, not more than 0.5% of ethanol), loss on drying (gravimetric method, not more than 5%), total ash (gravimetric method, not more than 5%), heavy metals (not more than 0.0002), microbiological purity and quantitative determination of the active substances (total polyphenols per pyrogallol, not less than 5% and 10%, respectively) [36].

### 3.3. Analysis of the Chemical Composition of the DECV

#### 3.3.1. Analysis of Hydroxycinnamic Acids, Flavonoids and Tannins

Analysis of hydroxycinnamic acids, flavonoids and tannin metabolites was performed by the method of HPLC using a reversed-phase column. The studies were carried out using a Discovery C18 chromatography column 250 × 4.6 mm with a silica gel sorbent modified with octadecyl groups. As a mobile phase we used 0.005 N orthophosphoric acid (eluent A) and acetonitrile (eluent B). The following chromatographic analysis parameters were used for the separation of hydroxycinnamic acids: Maximum feed speed of the mobile phase—0.7 mL/min, temperature of the column thermostat—25 °C, volume of the sample—5–10 μL, time of chromatography—50 min. Gradient elution mode—0 min 5% “B”, 8 min 8% “B”, 15 min 10% “B”, 30 min 20% “B”, 40 min 40% “B”, 41–42 min 75% “B”, 43–50 min 5% “B”. Scan time—0.6 s, detection range—190–400 nm, wavelengths—320 and 330 nm. 

The following chromatographic analysis parameters were used for the separation of flavonoids: Maximum feed speed of the mobile phase—0.8 mL/min, temperature of the column thermostat—25 °C, volume of the sample—5–10 μL, time of chromatography—60 min. Gradient elution mode—0 min 12% “B”, 30 min 25% “B”, 33 min 25% “B”, 38 min 30% “B”, 40 min 40% “B”, 41 min 80% “B”, 48 min 80% “B”, 49 min 12% “B”, 60 min 12% “B”. Scan time—0.6 s, detection range—190–400 nm, wavelength—340 nm. 

For the separation of the metabolites of tannins as the mobile phase, we used eluent A, a mixture of a 0.1% solution of trifluoroacetic acid, 5% solution of acetonitrile and deionized water; and for eluent B, a 0.1% solution of trifluoroacetic acid in acetonitrile. Chromatographic analysis parameters were maximum feed speed of the mobile phase—0.1 mL/min, temperature of the column thermostat—25 °C, volume of the sample—10 μL, time of chromatography—40 min. Gradient elution mode—0 min 0% “B”, 8 min 12% “B”, 10 min 12% “B”, 15 min 25% “B”, 20 min 25% “B”, 25 min 75% “B”, 28 min 75% “B”, 29 min 0% “B”, 40 min 0% “B”. Scan time—0.6 s, detection range—190–400 nm, wavelength—280 nm [37,38].

#### 3.3.2. Determination of the Quantitative Content of Different Groups of BAS

The quantitative content of the basic groups of BAS (total polyphenols, total hydroxycinnamic acids, tannins, total flavonoids and hydroquinone derivatives) in the DECV was determined by the method of absorption spectrophotometry on an SPh Cary-50 spectrophotometer [32,35,39,40]. 

Determination of the quantitative content of total polyphenols. The quantitative determination of the number of polyphenols per pyrogallol was performed by the method of the State Pharmacopoeia of Ukraine (SPhU) 2.0 [31,34].

Determination of the quantitative content of total flavonoids. The quantitative determination of the number of flavonoids per rutin was determined by a method based on the complexation reaction of flavonoids with aluminum chloride. The maximum absorption of the flavonoid complex by aluminum chloride was observed at a wavelength of 410 nm [32,35].

Determination of the quantitative content of hydroquinone derivatives. The quantitative determination of hydroquinone derivatives in the DECV was carried out by spectrophotometry according to the SPhU [35].

Determination of the quantitative content of hydroxycinnamic acid derivatives. The content of hydroxycinnamic acid derivatives per chlorogenic acid in the DECV was determined by spectrophotometry [32,35].

Determination of the quantitative content of tannins. The quantitative content of tannins per pyrogallol was determined by the method of SPhU 2.1 [35,39].

#### 3.3.3. Quantitative Analysis of Extracts

Quantitative determination of the hydrochloric acid derivatives, flavonoids and phenolic compounds in the filtrate in terms of dry residue was performed by a spectrophotometric method. The optical density was measured on a Specol 1500 spectrophotometer (Neuchâtel, Switzerland). The content of hydroxybutyric acid derivatives was determined in terms of chlorogenic acid at 327 nm, the content of the sum of flavonoids in terms of the absorbance at a wavelength of 417 nm after the formation of complexes with aluminum chloride, and the content of the sum of phenolic compounds in terms of gallic acid. For statistical validity, the experiments were performed at least five times [32,39,40].

### 3.4. Study of the Pharmacological Activities of DECV

The study of the pharmacological activities of DECV was carried out with the advisory assistance of different experts in the field (see Acknowledgements). Experimental work was carried out in the scope of simple pharmacological screening [41]. The experiments were carried out on white nonlinear mice and Wistar rats bred in the IFNMU vivarium, which were standardized by physiological and biochemical parameters and were kept under vivarium conditions in accordance with hygiene standards. All subjects gave their informed consent for inclusion before they participated in the study. The study was conducted in accordance with the Declaration of Helsinki, and the protocol was approved by the Ethics Committee of Ethics Commission of Ivano-Frankivsk Medical University (Projects “Pharmacognostic study of heather (*Calluna vulgaris* L. Hull.)”, № 98/17 dated 21/12/2017, “Studies of some wild and cultivated medicinal plants of the western region of Ukraine and development drugs based on them”, No state registration 0110U006205). The experiment was carried out in accordance with the International Principles of the European Convention for the Protection of Vertebrate Animals Used for Experimental and Other Scientific Purposes [42,43,44].

#### 3.4.1. Determination of Acute Toxicity

To determine the acute toxicity, the method of preclinical study of the safety of drugs was used. The animals were divided into 3 groups of 6 animals each. Animals of the first group were administered extract 1; animals of the second group extract 2; animals of the third group were the control. The animals were monitored for 14 days. The degree of the substance toxicity was evaluated on the basis of changes of the general condition of the animals, mortality, and the effect of the extracts on the dynamics of the body weight of the animals. The toxicity class was determined according to the general classification [41,45,46]. 

#### 3.4.2. Determination of Anti-Inflammatory Activity

The study of the anti-inflammatory activity of the DECV was carried out in accordance with the guidelines “Preclinical Studies of Drugs” [33,41,47]. The experiments were performed on male rats weighing 180–220 g that were divided into 5 groups of 6 animals each. The first group was the untreated model control, and the animals of the fifth group were the untreated non-modelled control. To study the effect of the DECV on the progression of the exudative phase of inflammation, a model of rat paw oedema caused by subplantar injection of a phlogogenic agent was used. To establish the model, 0.1 mL of a 2% aqueous formalin solution was injected under the hindpaw aponeurosis. Paw size was measured before and during the greatest development of oedema (5 h). Two hours before and immediately after the injection of the phlogogenic agent, the animals were administered the DECV intraperitoneally at a dose of 100 mg/kg. As a reference drug, we selected an herbal medicine, which is a standard with an established anti-inflammatory activity, commercial *Hypericum tincture* (Halychpharm Private Joint Stock Company, Lviv, PJSC Kyivmedpreparat, Kiev, Ukraine, drug and extract ratio 1:5, 40% ethanol, minimum 0.15% of total flavonoids, expressed as rutin). The reference drug was administered to the animals at an average therapeutic dose of 100 mg/kg. Measurement of the paw size was done oncometrically before the experiment, after 1 and 3 h and at the time of the maximum development of oedema (5 h). The effect of DECV was evaluated by its ability to suppress rat paw oedema.

#### 3.4.3. Determination of Antimicrobial Activity

The antimicrobial activity of DECV was studied by the method of diffusion of the active substances into agar using paper disks [16,36]. The concentration of the active substance on the disks was 5 mg. As universal nutrient media, 5% blood agar and daily broth cultures based on 1% sugar broth in a suspension of 1 billion microbial bodies were used. To do this, 1 mL of bacterial mixture was placed on the surface of 5% blood agar and rubbed evenly into it. The cultures were incubated at 37 °C for 24–72 h depending on the cultural characteristics of the studied organisms. Strains *Pseudomonas aeruginosa* ATCC 27853, *Escherichia coli* ATCC 25922, *Proteus vulgaris* ATCC 6896, *Staphylococcus aureus* ATCC 25923, and *Staphylococcus epidermidis* 14990 were obtained from the collection of cultures of microorganisms of the Department of Microbiology, Virology and Biotechnology ONU named I.I. Mechnikov, as well as from the laboratory of microbiology and immunology of the Institute of Gastroenterology of the National Academy of Medical Sciences of Ukraine. The activity of the DECV was determined by its ability to inhibit the growth of microorganisms around a paper disk saturated with the test substance. Ampicillin and oleandomycin at doses of 30 μg per disk were used as the reference substances.

#### 3.4.4. Determination of the Neurotropic Activity

Experiments to evaluate the neurotropic activity of the DECV were done on 24 white nonlinear adult male mice weighing 18–20 g bred in the IFNMU vivarium. Animals were divided into 4 groups of 6 animals each. The investigated water and hydroethanolic DECV were administered intraperitoneally at an effective dose of 50 mg/kg, and the reference drug, an extract of Valeriana officinalis root, was administered at the same dose. The extracts were dissolved in purified water. The administration of herbal extracts was done 4 days before the experiment and on the fouth day, 1 h before the experiment. Animals of the control group received an equivalent volume of purified water.

The experiments were conducted in an “Open Field” test that is one of the behavioral methods of studying the pharmacological activity of psychotropic agents [21,46]. Before the test, the animals were moved to a dark place and held there for 1 min. Then, the mouse was placed in the center of the arena with its back to the experimentalist. Using a digital video camera for 3 min, the following indicators were recorded: latent period of exit from the center of the arena; total motor activity; horizontal motor activity; appearance in the center of the arena; vertical motor activity represented by two types of stands: The hind legs of the animal remained on the arena floor and the front legs were placed against the wall (Climbing) or the front legs are in the air without any support (Rearing); grooming; the hole reflex or examination of holes in the arena floor; total time of fading (defined as the time when the animal does not move for more than 5 s); the number of defecation acts. Atypical behavioral responses to a stress factor were also recorded. Fading and the number of defecation acts were evaluated as a sign of fear in response to stress, and the intensity of these responses as a reflection of the emotional state of the animal.

#### 3.4.5. Determination of Anti-Anxiety and Antidepressant Effects

The studies to evaluate the anti-anxiety and antidepressant activity of the extracts were conducted in accordance with the “Guidelines for the experimental (preclinical) study of new pharmacological substances” [41,48]. Intact animals were involved in the experiment for morphological and anatomical study. Different tests can be differently sensitive to different doses of drugs, and the simulated anxiety and depression are heterogeneous, and that is why in our work the “Elevated Plus Maze”, “Porsolt Forced Swim Test” and “Cube” tests were used. These tests are behavioral methods of the study of the pharmacological activity of psychotropic agents [48].

The “Elevated Plus Maze” test, in which the behavior of animals is sensitive to the action of anxiotropic drugs, is one of the most commonly accepted tests for the evaluation of anxiety in rodents. The anxiolytic effect of the drug is determined by increasing the number of appearances of the animal in the open branches of the maze and the time of staying there without increasing the total motor activity [31,41,49].

The “Porsolt Forced Swim Test” (the behavioral despair test) is the basic model for the investigation of antidepressants. The animal is placed in a glass cylinder with a diameter of 20 cm and a height of 40 cm that is one-third filled with water with a temperature of 27 ± 1 °C. The duration of the experiment is 6 min. The latent period of the first “fading” (immobility for more than 5 s) and the total time of immobility were registered and interpreted as a manifestation of despair (depression). The duration of active swimming (vigorous movements with all of the paws and active swimming), passive swimming (weak movements of the paws needed just to keep the body afloat) and immobilization (absence of swimming movements) were also registered [41,50].

The “Cube” test is designed to investigate the cognitive response and the anxiety level. The wooden cube (3 cm^3^) was placed in the center of the cage (36 × 23 × 12 cm) with the mouse. The latent period of the first approach of the mouse to the cube, the number of approaches and the total time the mouse spent investigating the cube were registered. The test lasted for 5 min. According to the literature, the avoidance of an unfamiliar object in the home cage indicates an increased level of basic anxiety in mice. This is characteristic for both human and animal behavior [22,41,51].

### 3.5. Statistical Analysis

Statistical properties of random variables with an n-dimensional normal distribution are given by their correlation matrices, which can be calculated from the original matrices. Pharmacological research material was processed by the method of variational statistics with the calculation of the arithmetic mean and its standard error; the reliability of the compared values was estimated using the Student, Wilcoxon, and Mann–Whitney criteria with a probability level of ≤ 0.05 on a computer using Statistica 6.0 and Word Excel programs [35,52,53].

## 4. Conclusions

Nineteen phenolic substances were identified and quantified in the water and hydroethanolic extracts of *C. vulgaris*. Arbutin was dominant among the hydroquinone derivatives; chlorogenic acid among the hydroxycinnamic acids; rutin, hyperoside and quercetin-3-D-glucoside among the flavonoids; and (+)-gallocatechin and (-)-epigallocatechin among the tannin metabolites. The extract of *C. vulgaris* herb obtained with 70% hydroethanolic had the most pronounced anti-inflammatory, antimicrobial, anxiolytic, stress-protective, anti-anxiety and anti-depressant effects.

## Figures and Tables

**Table 1:** Chemical profile of dry extracts of *Calluna vulgaris* herb.

Substances	Content, %, *n* = 5	
	Water Extract	Hydroethanolic Extract
Phenols and their glycosides		
Arbutin	1.25 ± 0.05	0.83 ± 0.04
Methylarbutin	0.18 ± 0.03	0.23 ± 0.02
Hydroxycinnamic acids		
Chlorogenic	1.25 ± 0.03	1.74 ± 0.03
Caffeic	0.02 ± 0.01	0.03 ± 0.01
Ferulic	0.11 ± 0.02	0.12 ± 0.01
*p*-Coumaric	0.03 ± 0.01	0.04 ± 0.01
Flavonoids		
Rutin	0.65 ± 0.05	1.25 ± 0.05
Hyperoside	0.15 ± 0.05	0.2 ± 0.01
Quercetin-3-D-glucoside	0.17 ± 0.03	0.29 ± 0.01
Luteolin	0	0,05 ± 0.01
Apigenin	0	0,04 ± 0.01
Kaempferol	0.02 ± 0.01	0,05 ± 0.01
Tannins metabolites		
Gallic acid	0.07 ± 0.01	0.13 ± 0.01
(+)-Gallocatechin	0.21 ± 0.01	0.94 ± 0.02
(-)-Epigallocatechin	0.95 ± 0.05	1.36 ± 0.09
(+)-Catechin	0.13 ± 0.03	0.21 ± 0.03
(-)-Epicatechin	0.09 ± 0.01	0.26 ± 0.02
(-)-Catechin gallate	0.11 ± 0.02	0.24 ± 0.01
(-)Epicatechin gallate	0.05 ± 0.01	0.07 ± 0.01

**Table 2:** Quantitative content (%) of different groups of phenolic substances in the dry extracts of *Calluna vulgaris* herb.

The Group of BAS (Method of Analysis)	Content, $\bar{x}\pm \Delta \bar{x}$, *n* = 9	
	Water Extract	Hydroethanolic Extract
Total hydroquinone derivatives (spectrophotometry per arbutin)	10.51 ± 0.04	7.86 ± 0.03
Total hydroxycinnamic acids (spectrophotometry per chlorogenic acid)	5.87 ± 0.02	7.92 ± 0.04
Total flavonoids (spectrophotometry per rutin)	0.89 ± 0.02	2.33 ± 0.07
Total polyphenols (spectrophotometry per completely dry substance and pyrogallol)	5.95 ± 0.11	11.08 ± 0.20

**Table 3:** Indicators of the peripheral blood after the exudative phase of inflammation in the study of anti-inflammatory activity of *Calluna vulgaris* extracts.

Group of Animals	Indicators of Blood		
	Hemoglobin Content, g/L	Number of Red Blood Cells, ×10^12^/L	Number of Leukocytes, ×10^9^/L
Control	128.6 ± 2.5	7.25 ± 0.14	17.56 ± 0.38
Water extract	126.08 ± 3.6	6.98 ± 0.24	14.62 ± 0.46
Hydroethanolic extract	127.71 ± 1.5	6.79 ± 0.09	12.14 ± 0.27
*Hypericum* tincture	138.6 ± 3.7	7.12 ± 0.11	12.26 ± 0.16
Intact animals	131.86 ± 4.8	7.78 ± 0.15	11.92 ± 0.25

**Table 4:** Behavior of animals in conditions of the “Elevated Plus Maze” test.

Indicator	Number of Animals	Groups				
		Group I	Group II	Control	Group III	Intact Animals
1	2	3	4	5	6	7
Total number of appearances in the dark chamber	1	2 ± 0.24 *^,^**	1.14 ± 0.36 **	1.14	1.43 ± 0.42 *	1.71
	2	1.71 ± 0.20 **	1.43 ± 0.10 **	2.14	0.57 ± 0.04 *	1.71
	3	1.43 ± 0.29 *	2.29 ± 0.05 *	0.71	0.86 ± 0.71 *	1.43
	4	1.86 ± 0.14 *	1.29 ± 0.28 **	1.29	0.57 ± 0.41 *	1.86
	5	1.57 ± 0.36 *	1.29 ± 0.79 **	1.29	1.43 ± 1 *	1.14
	Σ 1–5	8.57 ± 0.31 **	7.43 ± 0.20 **	6.57	4.86 ± 0.29 *	7.86
Total time of staying in the dark branches of the maze, s	1	19 ± 0.01 **	18.14 ± 0.17 **	18.86	40.71 ± 0.10 *	45.57
	2	23.57 ± 0.09 **	18.43 ± 0.02 **	24.43	40.86 ± 0.17 *	45.57
	3	23.71 ± 0.02 **	33.43 ± 0.12 **	22.71	44.43 ± 0.24 *	51.86
	4	23.14 ± 0.02 **	32.71 ± 0.24 **	21.86	47.14 ± 0.11 *	46.14
	5	13.86 ± 0.02 **	33.57 ± 0.17 **	22.71	42.57 ± 0.04 *	54.57
	Σ 1–5	103.29 ± 0.02 **	136.29 ± 0.04 **	110.57	215.71 ± 0.09 *	243.71
Total number of appearances in the central location	1	2.86 ± 0.24 **	2.29 ± 0.58 *	2	2 ± 1 *	1.43
	2	3.14 ± 0.40 **	2.57 ± 0.34 **	2.86	1.86 ± 0.20 *	2.43
	3	2.14 ± 0.35 **	2.29 ± 0.27 **	2	1.14 ± 0.10 *	1.14
	4	3.43 ± 0.07 **	2 ± 0.60 *	2.14	1.14 ± 0.22 *	2.43
	5	2.57 ± 0.23 **	1.14 ± 0.20 *	2.29	1.72 ± 0.68 *	1
	Σ 1–5	14.14 ± 0.12 **	10.29 ± 0.49 **	11.29	7.86 ± 0.31 *	8.43
Total number of appearances in the illuminated branches of the maze	1	1.57 ± 0.22 **	1 ± 0.36 **	1.14	0.71 ± 0.10 *	0.86
	2	0.86 ± 0.46 **	0.86 ± 0.42 **	0.71	0.57 ± 0.59 *	0.14
	3	0.71 ± 0.41 **	0.71 ± 0.41 **	0.57	0.29 ± 0.36 *	0.43
	4	0.86 ± 0.09 **	0.43 ± 0.36 *^.^**	0.71	0.14 ± 0.17 *	0.71
	5	1.14 ± 0.06 **	0.29 ± 0.27 *	0.71	0.43 ± 0.42 *	0.14
	Σ 1–5	5.14 ± 0.04 **	3.29 ± 0.27 **	3.86	2.14 ± 0.07 *	2.29
Total time of staying in the illuminated branches of the maze, s	1	35.14 ± 0.02 **	34.71 ± 0.11 **	32.71	14.71 ± 0.11 *	7.14
	2	26.57 ± 0.20 **	36.29 ± 0.04 **	22.71	15 ± 0.60 *	6
	3	27.43 ± 0.01 **	20.14 ± 0.11 **	25.86	7.14 ± 0.08 *	3.86
	4	25.14 ± 0.07 **	19.43 ± 0.13 **	27.43	9.43 ± 0.24	8.86
	5	24.14 ± 0.11 **	18.29 ± 0.17 **	24.86	10.71 ± 0.11 *	0.86
	Σ 1–5	138.43 ± 0.04 **	128.86 ± 0.02 **	133.57	57 ± 0.11 *	26.71
Total time of staying in the central location, s	1	5.86 ± 0.86 **	7.14 ± 0.31 **	8.43	4.57 ± 1 *	7.29
	2	9.86 ± 0.15 **	5.29 ± 0.07 *	12.86	3.14 ± 0.07 *	8.43
	3	8.86 ± 0.73 **	6.43 ± 0.34 *	11.43	8.43 ± 0.46 *	4.29
	4	10.29 ± 0.12 **	7.86 ± 0.61 *	10.71	3.43 ± 0.17 *	5
	5	22 ± 0.06 **	8.14 ± 0.34 *	12.43	6.71 ± 0.17 *	4.57
	Σ 1–5	56.86 ± 0.12 **	34.86 ± 0.23 *	55.86	26.29 ± 0.23 *	29.57
Number of cases of looking down (risk assessment)	1	4.86 ± 0.09 **	2.57 ± 0.91 **	2.43	2.57 ± 1 *	1.29
	2	3.14 ± 0.40 *	3.29 ± 0.10 *	1.71	2.14 ± 0.78 *	0.57
	3	3.71 ± 0.23 *^/^**	1.57 ± 0.10 *	2.14	2.14 ± 0.89 *	0.43
	4	3.57 ± 0.02 **	1.29 ± 0.20 *	2.86	1.43 ± 0.34 *	0.71
	5	4.57 ± 0.04 **	1.71 ± 0.05 *	3.57	1.43 ± 0.07 *	0.29
	Σ 1–5	19.86 ± 0.02 **	10.43 ± 0.17 *	12.71	9.71 ± 0.75 *	3.29
The latent period of the first appearance in the branch of the maze		0.07 ± 0.34 **	0.36 ± 0.28 *	0.78	0.8 ± 0.79 *	1.52

Note: Group I—animals treated with hydroethanolic extract, group II—animals treated with water extract, group III—animals treated with *Hypericum tincture*, n—number of animals, *—the reliability of deviations in relation to the control group data, **—the reliability of deviations in relation to the reference group data (*p* ≤ 0.05).

**Table 5:** Behavior of animals in conditions of the “Porsolt Forced Swim Test”.

Groups of Animals		Latent Period of The First “Fading”	Active Swimming, s	Passive Swimming, s	Total Time of Immobility, s	Number of Dives
**Group I**	Day 1 of experiment	3.47 ± 0.46 *	190.29 ± 0.17 *	156 ± 0.86 *	13.71 ± 0.10 **	3.43 ± 0.06 **
Group II		3.23 ± 0.46 *	93.43 ± 0.12 **	217.71 ± 0.01 **	48.86 ± 0.11 *	2.43 ± 0.28 **
Group III		3.25 ± 0.39 *	136.14 ± 0.73 *	156.14 ± 0.73 *	67.71 ± 0.23 *	0.14 ± 0.10 *
Control		4.09	157.14	177	25.86	1.86
Intact animals		3.07	98	201	61	1.29
Group I	Day 3 of experiment	3.73 ± 0.75 *	99.57 ± 0.11 **	245.29 ± 0.55 **	15.14 ± 0.46 **	0.86
Group II		2.98 ± 0.49 **	83.29 ± 0.61 **	226.71 ± 0.17 *	50 ± 0.31 *	0
Group III		3.85 ± 0.61 *	58.71 ± 0.61 *	263.29 ± 0.86 *	38 ± 0.49 *	0
Control		3.4	71.14	263.43	25.43	0.14
Intact animals		2.4	78.86	141.57	107	2.43
Group I	Day 5 of experiment	2.89 ± 0.34 *	72.14 ± 0.17 *	215 ± 0.39 *	62.14 ± 0.61 **	0.57
Group II		3.26 ± 0.60 **^/^*	45.29 ± 0.02 *	260.14 ± 0.12 *	45 ± 0.34 **	0.14
Group III		2.22 ± 0.31 *	58.57 ± 0.02 *	215.14 ± 0.39 *	86.29 ± 0.17 *	0.57
Intact animals		3.15	34.14	297	43.14	0

Note: Group I—animals treated with hydroethanolic extract, group II—animals treated with water extract, group III—animals treated with *Hypericum tincture*, n—number of animals, *—the reliability of deviations in relation to the control group data, **—the reliability of deviations in relation to the reference group data (*p* ≤ 0.05).

**Table 6:** Behavior of animals in conditions of the “Cube” test.

The Investigated Indicator	Group I	Group II	Control	Group III	Intact Animals
Latent period of the first approach to the cube, s	0.46 ± 0.01 **	1.64 ± 0.46 *	2.73	1.73 ± 0.34 *	0.72
Total number of approaches to the cube	5.00 ± 0.07 *	3.71 ± 0.46 *	2.71	3.86 ± 0.27 *	6.71
Total time of the cube examination, s	9.43 ± 0.34 *	14.57 ± 0.02	4.43	7.43 ± 0.24 *	8.86
